# Bone-on-Bone Telescopic Fixation With Step-Plate Stabilization: A Novel Approach for Enhanced Stability in LeFort I Maxillary Advancement for Cleft-Related Hypoplasia

**DOI:** 10.7759/cureus.99939

**Published:** 2025-12-23

**Authors:** Mohammed Ali Hamid Syed, Samir Mansuri, Philip Mathew, Rahul VC Tiwari, P Vijay Anilkumar, Heena Dixit, Niti Dharmendra Shah, Seema Gupta, Manish Sharma

**Affiliations:** 1 Department of Orthodontics, Al Harkan Medical Center, Unaizah, SAU; 2 Department of Oral and Maxillofacial Surgery, Al Kuwait Hospital, Dubai, ARE; 3 Department of Oral and Maxillofacial Surgery, Jubilee Mission Medical College and Research Institute, Thrissur, IND; 4 Department of Oral and Maxillofacial Surgery, RKDF Dental College and Research Centre, Bhopal, IND; 5 Department of Oral and Maxillofacial Surgery, Sree Sai Dental College and Research Institute, Srikakulam, IND; 6 Department of Public Health, Commissionerate of Health and Family Welfare, Hyderabad, IND; 7 Department of Global Management, Thunderbird School of Global Management, Arizona State University (ASU), Phoenix, USA; 8 Department of Orthodontics, Kothiwal Dental College and Research Centre, Moradabad, IND; 9 Department of Oral Pathology, Jawahar Medical Foundation's Annasaheb Chudaman Patil Dental College, Dhule, IND

**Keywords:** advancement, cleft lip, cleft palate, maxillary hypoplasia, telescopic

## Abstract

Introduction: Patients with cleft lip and palate (CLP) frequently require secondary LeFort I maxillary advancement due to severe scar-induced relapse with conventional techniques. This study introduced and evaluated bone-on-bone telescopic fixation with step-plate stabilization as a geometry-dependent solution to improve long-term stability. The aim of this study was to assess the skeletal stability, relapse rates, esthetic outcomes, and complications of the novel bone-on-bone telescopic LeFort I advancement technique in cleft maxillary hypoplasia.

Materials and methods: A retrospective analysis of 90 consecutive non-syndromic CLP patients (52 females, 38 males; mean age 21.3 ± 3.8 years) who underwent LeFort I advancement using telescopic bone overlap and custom step-plate fixation between 2021 and 2022 was conducted. The minimum follow-up duration was 12 months. Horizontal and vertical stability was measured cephalometrically preoperatively (T0), immediately postoperatively (T1), and at ≥12-month (T2) intervals. A relapse >2 mm was considered clinically significant.

Results: The mean horizontal advancement was substantial and highly significant (p < .001). At T2, relapse occurred in only 18 patients (20%); 14 patients (15%) showed <2 mm horizontal relapse, and only four patients (5%) showed >2 mm horizontal relapse. The vertical position remained stable (p = 1.0). The maxillary position and maxillomandibular relationship improved significantly and were largely maintained (p < .001). Facial aesthetic scores (FAS) increased from 4.5 ± 1.05 to 7.4 ± 0.94 (p < .001; Cohen's d = 3.68). The incidence of complications was low (nerve disturbance, 7%; infection, 5%; reoperation, 6%). Bone grafting was required in 18 patients (20%). No significant correlation was found between the magnitude of advancement and relapse (r = −0.36, p = 0.121).

Conclusion: The bone-on-bone telescopic technique with step-plate fixation provided superior skeletal stability, minimal clinically relevant relapse, excellent esthetic improvement, and a favorable safety profile, establishing it as a reliable and minimally invasive option for the definitive correction of cleft maxillary hypoplasia.

## Introduction

Cleft lip and palate (CLP) remains one of the most prevalent congenital craniofacial deformities, affecting approximately one in 700 live births worldwide [1]. Despite early surgical repair, nearly half of the affected individuals develop maxillary hypoplasia during adolescence, necessitating secondary orthognathic correction [2]. LeFort I maxillary advancement osteotomy is widely regarded as the most reliable surgical approach for restoring sagittal and vertical maxillary dimensions in these patients [3]. However, postoperative relapse continues to pose a considerable clinical challenge, compromising occlusal stability, esthetic outcomes, and long-term patient satisfaction. Reported relapse rates vary substantially, with horizontal setback ranging from 17 to 20% and vertical relapse approaching 30-40%, largely attributed to scar tissue contraction, unfavorable soft-tissue vectors, compromised bony interfaces, and insufficient biomechanical fixation systems [4,5].

Although various modifications, including rigid internal fixation, bone grafting, and distraction osteogenesis, have been proposed to enhance stability, the fundamental biomechanical issue of posterior displacement is driven by cleft-associated scar tension [6]. Additionally, routine bone grafting increases surgical morbidity and cost, whereas patient-specific implants may not fully counteract scar-related forces in severe deformities [7]. Therefore, there is a critical need for a fixation technique that provides mechanical resistance to relapse and biological support for predictable bone healing.

The bone-on-bone telescopic technique with step-plate fixation, recently developed and patented, introduces a geometric overlap between the mobilized maxilla and the stable cranial segment, redefining stability from fixation-dependent to geometry-dependent. By mechanically preventing posterior displacement and promoting natural bone formation at the periosteal interface, this technique is a promising alternative to conventional fixation strategies. This study aimed to evaluate the clinical effectiveness of the bone-on-bone telescopic fixation technique in patients with cleft maxillary hypoplasia who underwent LeFort I advancement. The objectives were to (1) assess skeletal stability and relapse rates, (2) determine esthetic and patient-reported outcomes, (3) correlate the extent of maxillary advancement and relapse, and (4) document complications.

## Materials and methods

Study design and setting

This retrospective study was conducted in the Department of Oral and Maxillofacial Surgery. The study included patients who underwent LeFort I maxillary advancement using the bone-on-bone telescopic technique with step-plate fixation. This study evaluated 90 consecutive CLP patients operated on between January 2021 and December 2022, with postoperative follow-up extending to a minimum of 12 months. Ethical approval was obtained from the Institutional Ethics Committee of RKDF Dental College and Research Centre (RKDF/DC/2023/92). All patients provided informed consent for the procedure and analysis of anonymized records.

Patient selection

To ensure homogeneity, patients were selected based on predefined inclusion and exclusion criteria. The inclusion criteria comprised individuals aged 18-35 years with complete craniofacial growth, confirmed unilateral or bilateral CLP-associated maxillary hypoplasia, and an indication for maxillary advancement of ≥4 mm horizontally or ≥3 mm vertically. All patients had completed the presurgical orthodontic preparation and had complete clinical and radiographic records for the required follow-up duration. The exclusion criteria included active infection, significant systemic illness, previous orthognathic surgery, syndromic clefts or complex craniofacial anomalies, pregnancy or lactation, psychological contraindications to informed consent, and incomplete radiographic or clinical data.

Sample size estimation

The required sample size was calculated using the G*Power software (version 3.1.9.2; Heinrich Heine University, Düsseldorf, Germany). The sample size was determined a priori using a power analysis based on the primary outcome of skeletal relapse. The calculation was based on a reported mean relapse magnitude of 1.8 mm with a standard deviation of 0.9 mm following maxillary advancement [8]. To detect a clinically relevant difference of 1.0 mm with 80% statistical power and a two-sided alpha error of 5%, a minimum of 86 patients was required. The final target enrollment was set at 90 patients.

Preoperative assessment

Preoperative evaluation included detailed clinical history, clinical examination, facial esthetic assessment, dental occlusion analysis, and temporomandibular joint evaluation, as documented in the patients' clinical case records. Radiographic records involved lateral cephalograms, panoramic radiographs, and cone-beam computed tomography (CBCT) when present. Standard cephalometric parameters were measured using the Dolphin Imaging software (Dolphin Imaging and Management Solutions, Chatsworth, CA) (Table 1).

**Table 1:** Cephalometric landmarks used in the study.

S No.	Variables	Description
1.	SNA angle (degrees)	An angle between SN and NA lines where S (Sella) is the middle point of Sella turcica (S) and N (Nasion) is the anterior-most point on the frontonasal suture. A point is the deepest point in the anterior concavity of the maxilla. It suggests the position of the maxilla with respect to the cranial base.
2.	ANB angle (degrees)	An angle between the NA and NB lines where the B point is the deepest point in the anterior concavity of the mandible. It suggests the maxilla-mandibular position.
3.	N perpendicular to A point as the linear distance of the position of the maxilla (mm)	The linear distance of point A to a line perpendicular to Frankfort's horizontal plane (a plane joining the superior point of the external auditory meatus and the lowermost point on the bony orbit) from point N. It suggests the horizontal position of the maxilla.
4.	Vertical position of the maxilla (mm)	The vertical linear distance of N to ANS (anterior nasal spine). It suggests the vertical position of the maxilla.

**Figure 1:**
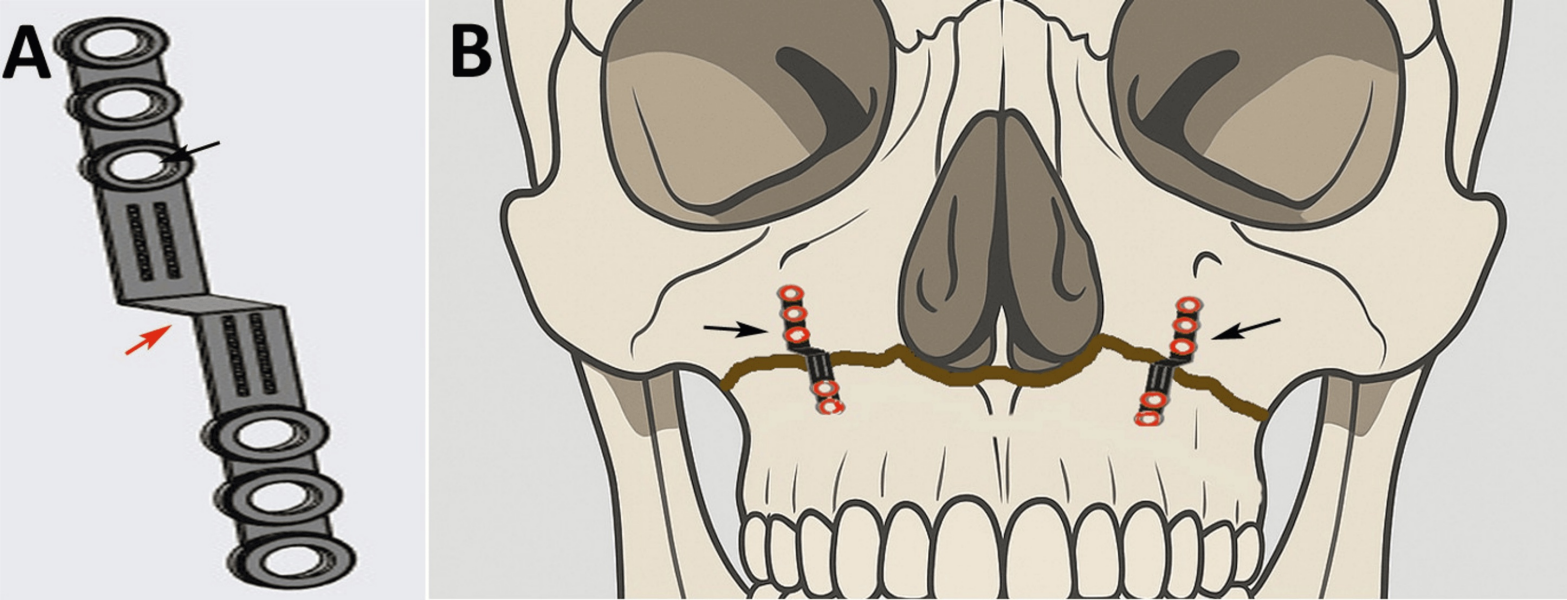
(A) Custom-designed step metal plate featuring multiple screw fixation holes. The step configuration (red arrow) and plate arms (black arrow) allow stabilization across displaced maxillary segments. (B) Schematic illustration demonstrating bilateral application of the step plates along the Le Fort I fracture line. The plates (black arrows) are positioned to bridge the fracture and provide stable fixation of the maxillary segments. This image was created by Philip Mathew and Rahul Tiwari.

Baseline facial photographs (frontal, lateral, oblique, and smiling views) were recorded using standardized protocols. Facial aesthetic scores (FAS, 0-10 scale) were independently assigned by two calibrated evaluators [9]. The FAS is free to use for research purposes. All patients underwent pre-surgical orthodontic alignment to establish optimal occlusion and dental decompensation prior to surgery. A patented fixation plate (design patent no. 454767-001, Indian Patent Registry, dated 24/07/2025) was designed for this surgery (Figure 1). The patented fixation system consisted of a custom titanium step-plate (Ti-6Al-4V) with a thickness of 1.5-2.0 mm and a step height of 2-3 mm, corresponding to the planned telescopic bony overlap. The overall plate length ranged from 18 to 22 mm, with two screw holes on each side of the step to engage both the stable cranial segment and the advanced maxillary segment. Fixation was achieved using 2.0-mm diameter monocortical titanium screws of 6-8 mm length; superior screws were placed with a posterosuperior vector into the stable maxillary buttress, while inferior screws were directed anteroinferiorly into the mobilized segment to counter posterior relapse and rotational forces. Intraoperative advancement was verified using calibrated surgical calipers and confirmed against the intermediate occlusal splint, ensuring a bilateral telescopic overlap of 2-3 mm prior to final fixation. Bone grafting was performed selectively only when residual gaps exceeded 3 mm, posterior bony contact was inadequate after telescoping, or segment stability was insufficient following fixation; minor gaps were left ungrafted to permit spontaneous bone healing. Preoperative planning was performed using lateral cephalograms and CBCT imported into Dolphin Imaging software, with standard landmarks (Sella, Nasion, Point A, ANS, PNS, and occlusal plane) used for virtual maxillary repositioning and splint fabrication. Digital cephalometric tracings were performed at T0, T1, and T2 by two blinded examiners using standardized magnification, and inter- and intra-examiner reliability was assessed using intraclass correlation coefficients, with values ≥0.85 considered acceptable.

Surgical technique: bone-on-bone telescopic advancement with step-plate fixation

**Figure 2:**
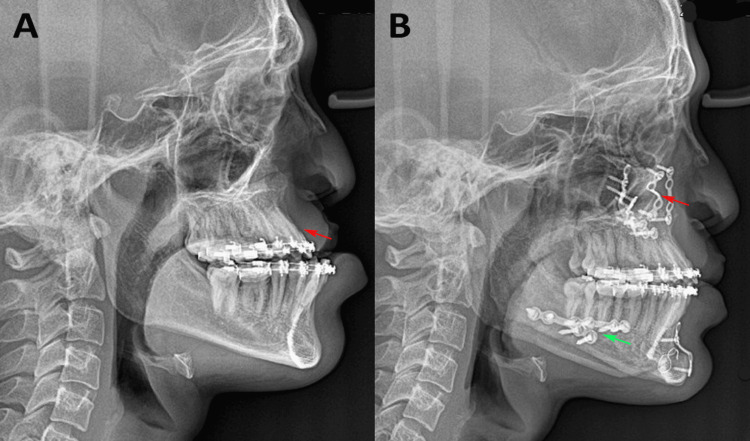
Bone-on-bone telescopic fixation technique. (A) Preoperative lateral cephalometric radiograph of a cleft lip and palate patient with maxillary hypoplasia and mandibular prognathism prior to surgery (red arrow indicating the maxillary hypoplasia); (B) postoperative lateral cephalometric radiograph demonstrating the bone-on-bone telescopic fixation technique following maxillary advancement using a step plate as visible at the maxillary osteotomy site (red arrow), along with mandibular setback surgery (green arrow). Original images of the patient, used with the patient's permission.

All surgeries were performed under general anesthesia by the same maxillofacial team to ensure technical consistency. A maxillary vestibular incision provided access, followed by subperiosteal elevation and a quadrangular or high LeFort I osteotomy, maintaining safe margins from the tooth apices and neurovascular structures. Downfracture was achieved after pterygomaxillary disjunction and septal osteotomy. Following mobilization, the maxilla was repositioned according to the planned movements, and the innovative bone-on-bone technique was applied. Unlike conventional end-to-end positioning, the superior segment overlaps the mobilized segment by 2-3 mm, creating a telescopic bone interface. This geometric overlap served as a mechanical stop against posterior relapse. Custom-designed titanium step plates (1.5-2.0 mm) were positioned bilaterally on the zygomatic buttress. The step configuration allowed secure fixation across both segments with optimal screw vectors. Bone grafting was reserved only for severe deficiencies requiring additional structural support. The wounds were closed in layers, and occlusion was reversed before extubation (Figure 2).

Postoperative care and follow-up protocol

Postoperative management included prophylactic antibiotics, analgesics, anti-inflammatory medications, and chlorhexidine rinse. Patients were maintained on a liquid-to-soft diet for six weeks. Light-guiding elastics were used for two weeks, followed by progressive removal and occlusal refinement for up to six weeks. Scheduled follow-ups were performed at 1, 2, 4, 6, and 12 weeks, and subsequently at 3, 6, and 12 months. Clinical assessment focused on occlusion, wound healing, facial symmetry, and sensory changes. Serial lateral cephalograms were obtained immediately after surgery and at 12 months postoperatively. The cephalometric parameters used in the present study are listed in Table 1.

Outcome measures

The primary outcome assessment was skeletal stability and relapse. Cephalometric measurements were performed by two independent, blinded evaluators. Horizontal and vertical changes were recorded at each time point (preoperative as T0, immediate postoperative as T1, and at 12 months postoperative as T2). The percentage of relapse was calculated relative to that of the original surgical movement. Stability was further evaluated using changes in the position of point A. Secondary measures included FAS using a validated 0-10 scale; soft tissue adaptation, including lip support, nasolabial angle, and incisor exposure; intraoperative stability, documented by the surgical team; and complications, including nerve disturbance, malocclusion, infection, hardware issues, and need for grafting. All outcomes were recorded using standardized forms.

Statistical analysis

Statistical analysis was performed using IBM SPSS Statistics for Windows, Version 25 (Released 2017; IBM Corp., Armonk, New York). Descriptive statistics (mean ± standard deviation, frequency, and percentage) were used to summarize demographic and baseline clinical characteristics. The primary outcome of skeletal stability was assessed using the paired t-test. The relapse rate was reported as the frequency of patients with >2 mm horizontal movement. Changes in cephalometric parameters across preoperative, immediate, and 12-month time points were analyzed using repeated-measures analysis of variance (ANOVA). The relationship between the magnitude of surgical advancement and the degree of relapse was examined using Pearson's correlation coefficient. Secondary outcomes, including improvements in FAS scores, were analyzed using paired t-tests and descriptive statistics. Statistical significance was set at p < 0.05.

## Results

The sample consisted of 52 (57.77%) females and 38 (42.23%) males with a mean age of 21.3 ± 3.8 years and a predominance of bilateral CLP cases. Graft utilization was limited to 18 patients (20%). Relapse was observed in 18 patients (20%), which was statistically significant (P = 0.007). Complication rates were notably low, with nerve disturbance, infection, and reoperation each occurring in only five to nine (5-10%) of cases, which were statistically significant (p = 0.001), indicating that these adverse events were rare yet discernible from chance. The analysis inferred that the surgical protocol was associated with a controlled and predictable safety profile, although a notable minority of patients experienced skeletal relapse. The significant p-values for relapse and specific complications, despite their low incidence, highlighted important clinical endpoints for postoperative monitoring (Table 2).

**Table 2:** Demographic and clinical characteristics of the study cohort (one-sample χ² test against expected equal distribution or chance). *p < 0.001 denotes high statistical significance. Values are presented as frequency (n) and percentage (%). CLP: cleft lip and palate

Parameters	Categories	n	%	Chi Stats	p-value
Sex	Male	38	42.20	2.18	0.140
	Female	52	57.78		
CLP type	Unilateral	36	40.00	3.60	0.058
	Bilateral	54	60.00		
Graft used	Yes	18	20.00	32.40	0.001*
	No	72	80.00		
Relapse	Yes	18	20.00	32.40	0.001*
	No	72	80.00		
Nerve disturbances	Yes	5	5.50	71.11	0.001*
	No	85	94.50		
Infection	Yes	9	10.00	57.60	0.001*
	No	81	90.00		
Reoperation	Yes	5	5.50	71.11	0.001*
	No	85	94.50		

Repeated-measures ANOVA revealed highly significant changes over time for the SNA, ANB, and Point A positions (p < .001), confirming successful maxillary advancement. The immediate postoperative measurements showed substantial improvement, which was partially maintained at 12 months, although a slight, yet statistically significant relapse occurred. Notably, the vertical change in the maxillary position remained completely stable with no significant change (p = 1). These results suggest that the bone-on-bone telescopic advancement technique effectively achieved and maintained significant horizontal correction but demonstrated a measurable, though limited, tendency for relapse, highlighting the importance of the technique's mechanical stop while underscoring the need for ongoing stability monitoring (Table 3).

**Table 3:** Cephalometric changes over time assessed by repeated-measures analysis of variance (ANOVA). *p < 0.001 denotes highly statistically significant values using the post-hoc Bonferroni test. Values are presented as mean ± standard deviation (SD).

Parameters	Preoperative as T0 (Mean ± SD)	Immediate Postoperative as T1 (Mean ± SD)	12-Month Postoperative as T2 (Mean ± SD)	F Stats	p-value
SNA angle (degrees)	77.3 ± 1.59	82.94 ± 1.96	81.92 ± 2.01	260.84	0.001*
ANB angle (degrees)	-0.15 ± 0.99	5.49 ± 1.38	4.47 ± 1.54	242.34	0.001*
Point A linear distance (mm)	-3.1 ± 1.07	3.95 ± 1.85	2.68 ± 2.14	250.58	0.001*
Vertical position of maxilla (mm)	49.85 ± 1.98	49.85 ± 1.98	49.85 ± 1.98	0	1

The comparison of horizontal changes in the position of the maxilla from T0 to T2 revealed a highly significant mean relapse of 5.77 mm (t = -13.54, p < .001). These results demonstrate that while the bone-on-bone telescopic technique achieved considerable initial advancement, a significant skeletal relapse occurred within the first postoperative year, characterized by backward movement of the maxilla (Table 4).

**Table 4:** Net horizontal skeletal advancement from preoperative to ≥12-month postoperative. *p < 0.001 denotes highly statistically significant values using a paired t-test. df: degree of freedom

Variable	Mean Difference (T2-T0) (mm)	T Stats	df	p-value	Cohen's d
Net horizontal advancement of Point A (mm)	-5.77	-13.54	19	0.001*	3.03

A total of 18 (20%) patients relapsed. In terms of clinical relevance, 14 (15%) patients showed a relapse of less than 2 mm, whereas four (5%) patients showed a relapse of more than 2 mm with statistical significance (p < .001), as shown in Table 5.

**Table 5:** Magnitude of horizontal relapse in the 18 patients who experienced relapse. *p < 0.001 denotes highly statistically significant values using a paired t-test. The sample is presented as frequency (n) and percentage (%). Relapse is presented as mean and standard deviation.

Relapse Category	18 (20%)	Mean	Standard Deviation	T Stats	p-value	Cohen's d
<2 mm (clinically insignificant)	14 (15.6%)	0.96	0.31	-9.36	0.001*	5.23
>2 mm (clinically significant)	4 (4.4%)	2.53	0.25			

The FAS [9] demonstrated a highly significant improvement following surgery, increasing from a mean of 4.5 ± 1.05 at T0 to a mean of 7.4 ± 0.94 at T2 (t = -16.46, p < .001). The exceptionally large Cohen's d effect size of 3.68 confirms the change is both statistically robust and clinically substantial. These results indicate that the maxillary advancement procedure produced marked and consistent enhancement in facial esthetics (Table 6).

**Table 6:** Comparison of facial aesthetic scores (FAS). *p < 0.001 denotes highly statistically significant values using a paired t-test. Values are presented as mean ± standard deviation (SD). CI: confidence interval

Variables	95% CI for Mean	Mean ± SD	T Stats	p-value	Cohen's d
Preoperative (FAS scores)	4.01–4.99	4.50 ± 1.05	-16.46	0.001*	3.68
Postoperative at 12 months (FAS scores)	6.96–7.84	7.40 ± 0.94			

A weak negative correlation was observed between the magnitude of surgical advancement and subsequent skeletal relapse (r = -0.36). However, this relationship was not statistically significant (p = 0.121). This indicates that within this cohort, the amount of maxillary advancement did not reliably predict the degree of relapse (Table 7).

**Table 7:** Pearson correlation between the magnitude of surgical advancement and horizontal relapse. p > 0.05 denotes no statistical significance. CI: confidence interval

Correlation Between Advancement and Relapse	r-value	p-value	95% CI
Actual advancement (mm) and Relapse magnitude (mm)	-0.36	0.121	-0.69–0.10

## Discussion

This study evaluated the clinical efficacy of the bone-on-bone telescopic fixation technique with step-plate fixation for LeFort I maxillary advancement in 90 patients with CLP-associated maxillary hypoplasia. Key findings included a 20% relapse rate, with the majority (15%) experiencing less than 2 mm of horizontal relapse and only 5% exceeding 2 mm, alongside significant improvements in cephalometric parameters and FAS. Complications were minimal, occurring in 5-10% of cases, and no significant correlation was found between advancement magnitude and relapse. These results suggest that the telescopic technique provides enhanced mechanical stability compared with traditional methods, potentially addressing the persistent challenge of postoperative relapse in CLP orthognathic surgery.

Skeletal stability is the primary strength of this approach. The observed 20% relapse rate aligns with the reported ranges in conventional LeFort I osteotomies for CLP patients, where horizontal relapse typically varies from 17 to 30% [4,10,11]. Wangsrimongkol et al. [10] reported a mean relapse of 1-1.5 mm at 12-month follow-up in 51 CLP patients treated with LeFort I maxillary advancement. For instance, a systematic review by Saltaji et al. [11] documented long-term horizontal relapse of 20-30% at Point A following maxillary advancement in four studies and 30-40% in three studies following conventional LeFort I osteotomy in CLP patients, attributing it to scar tension and soft-tissue forces. Similarly, da Silva et al. [12] reported vertical relapse at the two-year follow-up in patients with CLP treated with conventional LeFort I osteotomy.

In contrast, our study demonstrated vertical stability with no significant change, likely because of the geometric overlap in the telescopic design, which acts as a mechanical barrier against posterior and superior displacement. The mean net horizontal advancement from T0 to T2 was 5.77 mm, with relapse being predominantly minor (<2 mm in 15% of cases), supporting the technique's ability to mitigate relapse more effectively than rigid fixation alone. This is consistent with Valls-Ontañón et al. [5], who found that modifications enhancing bony contact reduced relapse by 10-15% in cleft populations. They further concluded that relapse was not related to bone grafting, overcorrection, or fixation type.

Our results further indicated that the observed relapse was not significantly correlated with the amount of maxillary advancement. Similar findings were reported by Watts et al. [13] for patients with unilateral CLP. Kumari et al. [14] performed step osteotomy for maxillary advancement in CLP patients and reported 21.63% and 41.54% relapse in the horizontal and vertical directions, respectively. The significantly lower relapse rate observed in our study favored the use of the bone-on-bone telescopic technique for maxillary advancement in patients with CLP. This discrepancy may stem from the biomechanical advantage of telescopic overlap, distributing forces more evenly and promoting periosteal bone healing without routine grafting, which was used in only 20% of our cases.

Esthetic and patient-reported outcomes were notably positive, with FAS improving from 4.5 to 7.4, indicating substantial clinical enhancement. This mirrors the findings of Andersen et al. [15], who reported high levels of patient satisfaction after maxillary advancement surgery in patients with CLP, emphasizing the role of maxillary repositioning in nasolabial harmony and lip support. Austin et al. [16] reported high levels of patient satisfaction with LeFort I osteotomy.

The results of this study indicated that the complication rates were low. Similar findings were reported in a previous study [17]. Hwang et al. [18] treated 17 patients with CLP and reported that two patients had inadequate fractures, three patients had injury to the inferior alveolar nerve, and one patient had a fistula. This safety profile supports the minimally invasive nature of the technique, as bone grafting is selective and reduces morbidity compared with routine autologous grafting protocols, which increase operative time and donor-site complications.

Clinical implications

The bone-on-bone telescopic technique offers a viable alternative for CLP maxillary hypoplasia, particularly in moderate-to-severe cases requiring ≥4 mm advancement. Shifting stability from fixation-dependent to geometry-dependent may reduce the need for adjuncts such as distraction osteogenesis, which, while effective (relapse <10%), prolongs treatment and increases costs. Clinicians should prioritize this method for patients with high scar tension and monitor relapse via serial cephalograms in the first year. Integration with presurgical orthodontics enhances outcomes and potentially improves long-term occlusal and esthetic stability.

Limitations

Several limitations should be emphasized when interpreting these findings. The retrospective, single-arm design without a contemporaneous control group limits causal inference and may overestimate treatment effects relative to conventional fixation methods. Skeletal stability was assessed using standardized two-dimensional cephalometry rather than routine three-dimensional CBCT, which may underestimate subtle transverse or rotational relapse. In addition, although consecutive cases were included, selection bias and attrition related to follow-up availability cannot be fully excluded, potentially limiting generalizability beyond similar tertiary cleft centers. Patient satisfaction and esthetic improvement observed in this cohort are broadly consistent with reported outcomes following orthognathic surgery in cleft and non-cleft populations, and the low complication rates should be interpreted within the known risk profile of orthognathic procedures reported in the literature.

## Conclusions

The bone-on-bone telescopic fixation technique with step-plate stabilization demonstrated substantial immediate skeletal correction, a low incidence of clinically significant relapse, and marked improvement in facial esthetics at 12-month follow-up. Although a measurable degree of backward skeletal movement was observed, the majority of relapses remained clinically minor, suggesting that geometric overlap may contribute to greater resistance to scar-related displacement. Within the limitations of a retrospective, non-comparative design, these findings indicate that the technique is a promising and biologically sound alternative for managing cleft-related maxillary hypoplasia. Prospective controlled studies incorporating validated patient-reported outcome measures are warranted to confirm its comparative effectiveness and long-term stability.
